# Patients and Surfaces: Integrated Clinical–Environmental Surveillance of MDR Gram-Negative Bacteria in Critical-Care Units (Karachi, 2024–2025)

**DOI:** 10.3390/microorganisms13122762

**Published:** 2025-12-04

**Authors:** Zeb Hussain, Fizza Farooqui, Aleeza Ibrahim, Samina Baig

**Affiliations:** 1 Dow Institute of Medical Technology, Dow University of Health Sciences, Karachi 74200, Pakistan; alizaarain2002@gmail.com (A.I.); samina.baig@duhs.edu.pk (S.B.); 2 Department of Microbiology, OMI Institute Hospital, Karachi 74200, Pakistan; fizza.jameel@gmail.com; 3 Microbiology Department, Sindh Infectious Diseases Hospital & Research Center, Dow University of Health Sciences, Karachi 74200, Pakistan

**Keywords:** drug resistance, *Acinetobacter baumannii*, bacterial, carbapenemases, intensive units care, *Klebsiella pneumoniae*

## Abstract

Carbapenem-resistant Gram-negative (CR-GN) pathogens pose a critical threat to patient outcomes in high-dependency and intensive care environments. This study aimed to delineate species prevalence, antimicrobial resistance phenotypes, carbapenemase genotypes, and clinical–environmental transmission dynamics across critical-care units. Cross-sectional surveillance was conducted in six ICUs and HDUs of a tertiary-care hospital in Karachi, Pakistan. We identified predominant species, quantified resistance patterns, and detected carbapenemase genes using PCR, exclusively on meropenem-resistant isolates. Network analysis highlighted high-centrality contamination hubs across ICUs and HDUs. *Acinetobacter baumannii* (36.7%) and *Klebsiella pneumoniae* (33.9%) were predominant, with 58% originating from environmental reservoirs. Meropenem non-susceptibility was 55% (60/109), and colistin non-susceptibility was 68.6% (35/51), based on standardized CLSI testing. ICU isolates exhibited significantly higher meropenem resistance than HDU isolates. Among carbapenem-resistant isolates, *bla*OXA-48-like (52.8%) and *bla*NDM (25%) were most prevalent. Network topology revealed ICU1 and HDU2 as high-centrality transmission nodes. These findings highlight pervasive environmental colonization and heightened antimicrobial pressure in ICUs, necessitating reinforced decontamination protocols, antimicrobial stewardship, and continuous molecular surveillance. This study provides the first integrated clinical–environmental surveillance of MDR Gram-negative bacteria in Pakistan, revealing that over half of isolates originated from surfaces and that network-based mapping can pinpoint contamination hubs driving hospital transmission.

## 1. Introduction

The global spread of multidrug-resistant (MDR) Gram-negative bacteria threatens to reverse decades of progress in critical-care medicine [1,2]. The World Health Organization lists carbapenem-resistant *A. baumannii*, *P. aeruginosa*, and Enterobacterales as “critical priority” pathogens [3]. Infections caused by these organisms are associated with high mortality, prolonged hospitalization, and limited therapeutic options [4]. Carbapenemase genes—most notably *bla*OXA-48-like, *bla*NDM, *bla*VIM, and *bla*KPC—let bacteria hydrolyse carbapenems and spread readily via plasmids [5]. Surveillance in high-income countries reveals shifting epidemiology. *bla*OXA-48-like enzymes are class D β-lactamases with weak carbapenem hydrolysis. *bla*NDMs, by contrast, are metallo-β-lactamases that hydrolyze a broad spectrum of carbapenems. *bla*VIM enzymes are integron-associated metallo-β-lactamases, distinctively common in *Pseudomonas* and Enterobacterales [6,7]. New York City reported a seven-fold increase in *bla*NDM-positive carbapenem-resistant Enterobacterales (CRE) from 2019 to 2024. Epidemiological data from the US and UK between 2019–2025 show rising trends of *bla*NDM- and *bla*OXA-48-like carbapenemases, supported by recent CDC and UKHSA surveillance reports (2024–2025) [8,9]. The United Kingdom’s quarterly update showed *bla*NDM accounted for 37% of carbapenemase families between Q3 2024 and Q2 2025. This slightly exceeded *bla*OXA-48-like (35.7%) and greatly outnumbered *bla*KPC (18.4%) [10,11]. These trends are supported by recent reports from CDC (MMWR 2025) and UKHSA (2024–2025), which confirm the expansion of *bla*NDM- and *bla*OXA-48-like producers in healthcare settings [9]. Epidemiological transitions are well documented in high-income countries, but in South Asia, data are incomplete and underreported [12]. Low- and middle-income countries like Pakistan face heightened risks—high antibiotic use, limited diagnostic resources, and weak infection-control measures. Surveillance and molecular characterization studies are essential to quantify the local antimicrobial resistance burden and understand its contribution to the global crisis [13]. Recent national surveillance shows Pakistani ICUs have a high burden of carbapenem-resistant Enterobacterales and non-fermenters, with resistance rates often over 50–70% in tertiary-care settings. *bla*NDM and *bla*OXA-48-like carbapenemases predominate among clinical isolates. MDR *A. baumannii* is widespread in ventilated and device-dependent patients. Environmental sampling from Pakistani ICUs shows MDR Gram-negative organisms persist on high-touch surfaces, leading to ongoing contamination and transmission [14]. Critical-care environments harbor complex microbial communities where high-touch surfaces, invasive devices, and antibiotic pressure facilitate colonization by MDR Gram-negative organisms [15].

Despite these international signals, Pakistan’s critical-care units face distinct pressures—high antibiotic consumption, constrained diagnostics, and challenging infection-prevention capacity—yet integrated clinical-and-environmental surveillance remains limited [16,17]. Prior reports suggest *bla*OXA-48-like enzymes have been common locally, and *bla*NDM has emerged in parts of South Asia [18,19]. However, ward-level co-circulation across patients and high-touch surfaces, and the distribution of resistance genes between ICU and HDU settings, remain poorly characterized [15]. We therefore conducted a cross-sectional study (June 2024–June 2025) with the following objectives: (i) to quantify species and resistance patterns in critical-care wards, (ii) to compare the burden between ICU and HDU units, and (iii) to map clinical–environmental overlap and profile key carbapenemase/virulence genes to support infection-control priorities in Pakistan. Intensive care and high-dependency units are highly vulnerable to MDR Gram-negative pathogens because patients often need invasive procedures, mechanical ventilation, and broad-spectrum antibiotics [20]. Cross-transmission results from contaminated hands of healthcare workers, high-touch surfaces, and shared equipment [21]. Few studies combine clinical and environmental surveillance with molecular detection and network analysis to map transmission dynamics [22]. Therefore, this study aimed to characterize the prevalence, antimicrobial resistance, and gene profiles of MDR Gram-negative bacteria in critical-care wards, integrating clinical and environmental data.

## 2. Materials and Methods

### 2.1. Study Design and Setting

This cross-sectional observational study was conducted from June 2024 to June 2025 at the Sindh Infectious Diseases Hospital in Karachi, Pakistan, and included six critical-care wards: three ICUs (ICU1–ICU3) and three HDUs (HDU1–HDU3). In total, 109 non-duplicate Gram-negative isolates were obtained from 25 patients and their surrounding environments. Sample size calculations assumed a 50% resistance rate and targeted 80% power at the 0.05 significance level [23].

#### Sample Collection and Microbiological Identification

Clinical specimens, including sputum, tracheal aspirate, and urine, were received in microbiology laboratory from admitted patients in above mentioned six wards. Pathogens identified as multidrug-resistant (MDR) in culture, environmental swabs were then obtained from five high-touch surfaces, both clinical and environmental MDR isolates were included in the analysis. Multidrug resistance (MDR) was defined as resistance to ≥3 antimicrobial classes according to CLSI M100 (2024) [24]. All samples were transported on ice and processed within one hour. Microorganisms were recovered on MacConkey and blood agar and identified using biochemical tests. Quality-control strains, *Escherichia coli* ATCC 25922 and *P. aeruginosa* ATCC 27853, were included in the analyses [25].

### 2.2. Study Population and Sampling Strategy

To clarify the studied patient group, a purposive, index-patient-based sampling method was employed. All patients admitted to ICUs/HDUs were routinely screened for multidrug-resistant (MDR) Gram-negative organisms based on clinical samples submitted to the microbiology laboratory. When an MDR Gram-negative-positive patient was confirmed (index case), environmental surveillance was initiated. Environmental swabs were collected from high-touch surfaces within a 1–2 m radius of the index patient, including bed rails, bedside lockers, multiparameter monitors, ventilators, IV poles, and infusion pumps. Duplicate samples from the same patient and site were excluded [26].

### 2.3. Sample Size Justification

To clarify the rationale for sampling, an initial sample size was calculated using the single population proportion formula (50% resistance rate, 95% confidence interval [α = 0.05], 80% power). Nevertheless, considering the unpredictable nature of MDR outbreaks and ethical/practical constraints, a pragmatic, event-driven sampling approach was adopted instead.

Therefore, The number of isolates analyzed (*n* = 109) reflects all non-duplicate MDR Gram-negative isolates obtained from every eligible index patient and their surrounding environment during the study period, rather than a statistically predetermined target.

### 2.4. Antimicrobial Susceptibility Testing

Susceptibility testing followed CLSI M100 (2024) criteria. Disk diffusion was used for all agents except colistin, which was tested by broth microdilution (ISO-standardized cation-adjusted Mueller–Hinton broth). We report susceptible (S), intermediate (I), and resistant (R) categories separately, primary analyses defined ‘non-susceptible’ as I + R. Per-agent denominators varied (Table 1 footnotes) due to plate availability and isolate viability [27].

Colistin MIC testing was performed only for isolates that were viable after storage and for which broth microdilution plates were available. This pragmatic selection followed CLSI recommendations, which allow targeted MIC testing when resource limitations exist.

### 2.5. Molecular Detection of Carbapenemase and Virulence Genes

PCR was restricted to 60/109 meropenem-resistant isolates due to resource prioritization and the study’s specific focus on carbapenem resistance mechanisms. Genomic DNA was extracted from meropenem-resistant isolates using the “Thermo Scientific™ GeneJET” (Waltham, MA, USA) [28]. To amplify target genes, polymerase chain reaction (PCR) was performed for *bla*NDM, *bla*OXA-48 like, *bla*VIM, and fimH using published primers [29]. The selected carbapenemase genes (*bla*NDM, *bla*OXA-48-like, *blaVIM*) represent the predominant mechanisms reported in Pakistan. fimH was included as a virulence/adhesion marker associated with environmental persistence and colonization potential

PCR conditions: initial denaturation 95 °C for 5 min; 35 cycles of 95 °C 30 s, annealing 52–58 °C 30 s, extension 72 °C 45 s; final extension 72 °C 7 min. Primer sequences and amplicon sizes are provided in Supplementary Table S1. Amplicons were then visualized on 1.5% agarose gels alongside positive and negative controls. Representative gel images of *bla*NDM, *bla*OXA-48, fimH, and *bla*VIM products are provided as Supplementary Figures.

### 2.6. Statistical Analysis and Network Modeling

The primary outcome was meropenem non-susceptibility, assessed using isolate-level logistic regression. Multivariable logistic regression included ward type, species, specimen source, and month, with robust standard errors clustered by ward. Predictors included ward type (ICU vs. HDU), specimen source (clinical vs. environmental), species, and month (June–August fixed effects). Robust standard errors accounted for ward clustering. Secondary outcomes were imipenem non-susceptibility and MDR (≥3 classes). Multiple secondary comparisons were adjusted using the Benjamini–Hochberg method with a false discovery rate of α = 0.05. The preliminary cross-tabulation consisted of descriptive contingency tables (ward × species × resistance profile) to assess distribution patterns prior to regression modeling.

## 3. Results

### 3.1. Distribution of Species and Sources

Of 109 isolates, 49 (45%) originated from HDUs and 60 (55%) from ICUs. Notably, environmental samples constituted 63% of HDU isolates and 53% of ICU isolates. Regarding species identified, *A. baumannii* (40/109; 36.7%) and *K. pneumoniae* (37/109; 33.9%) were the predominant species. *P. aeruginosa* (11%), *E. coli* (8.3%), and *Enterobacter* spp. (7.3%), *Stenotrophomonas maltophilia* (1.8%) and *Citrobacter* spp. (0.9%). The species distribution was similar between ward types (χ^2^ = 0.11, *p* = 0.74). (Table 2) enumerates organism counts by ward.

### 3.2. Antibiotic Resistance Patterns

**Figure 1:**
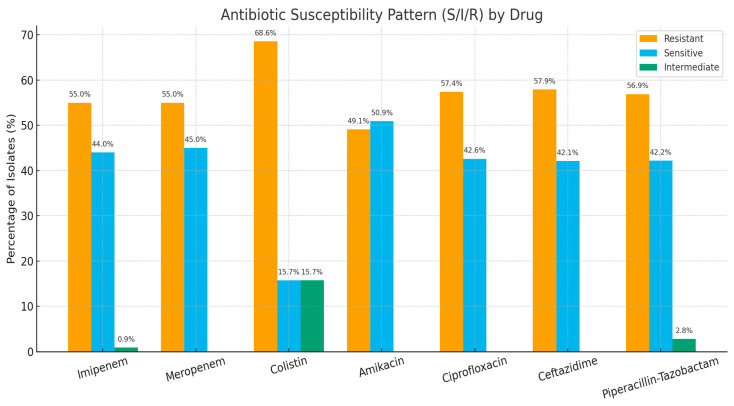
Antibiotic resistance and susceptibility profiles of isolates. Yellow bars represent resistant isolates and blue bars represent susceptible isolates across the eight antibiotics evaluated. Intermediate results are Green bars depicted in the figure.

Resistance to most antibiotics was high (Table 1). For example, 55% of isolates were resistant to imipenem and meropenem, 57.9% to ceftazidime, 57.4% to ciprofloxacin, and 56.9% to piperacillin–tazobactam. Amikacin resistance was observed in 49.1% of isolates. Among 51 isolates tested for colistin, 35 (68.6%) were resistant, 8 (15.7%) were intermediate, and 8 (15.7%) were susceptible (Figure 1). A chi-square test of independence was performed to compare resistance proportions across antibiotics. The association between antibiotic type and resistance was not statistically significant (χ^2^ = 5.73, df = 6, *p* = 0.454), indicating that resistance rates did not differ significantly between different antibiotics. Resistance proportions below treat I + R as ‘non-susceptible’ unless stated otherwise; full S/I/R breakdowns are shown in Table 1.

### 3.3. Predictors of Meropenem Resistance and Multidrug Resistance

Table 3 presents crude (unadjusted) logistic regression results assessing the association between ward type (ICU vs. HDU) and resistance to meropenem, imipenem, and multidrug resistance (MDR). For each outcome, the number of resistant isolates out of the total isolates in each ward is shown along with the odds ratio (ICU relative to HDU) and 95% confidence interval. Intermediate isolates were merged with sensitive isolates (S/I = 0, R = 1) to create a binary logistic outcome. Meropenem resistance was significantly higher among ICU isolates (65.0%) compared with HDU isolates (42.9%) with an odds ratio of 2.48 (95% CI: 1.14–5.38; *p* = 0.021). In contrast, imipenem resistance showed no significant difference between ICU and HDU isolates (OR = 1.00, 95% CI: 0.47–2.13; *p* = 0.993). Multidrug resistance was more common in ICU isolates (63.3% vs. 44.9%), with an odds ratio of 2.12 (95% CI: 0.98–4.58), but this did not reach statistical significance (*p* = 0.054), suggesting only a trend. Supplementary Table S1 provides the full logistic regression output, including coefficients, standard errors, odds ratios, confidence intervals, and model diagnostics.

Therefore, ward type appears to be a significant predictor for meropenem resistance but not for imipenem or MDR. These findings indicate that ICU settings may contribute to higher selective antibiotic pressure or greater exposure to resistant strains.

**Table 3:** Crude logistic regression for meropenem resistance, imipenem resistance and multidrug resistance (MDR) among ICU versus HDU isolates (*n* = 109). Breakpoints were interpreted according to CLSI M100 (2024), and non-susceptible was defined as intermediate or resistant versus susceptible. MDR was defined as non-susceptibility to ≥3 antibiotic classes.

Outcome	Predictor	ICU (R/Total)	HDU (R/Total)	Odds Ratio (95% CI)	Interpretation
Meropenem resistance	Ward (ICU vs. HDU)	39/60	21/49	2.48 (1.14–5.38)	Higher in ICU, significant
Imipenem resistance	Ward	33/60	27/49	1.00 (0.47–2.13)	No difference
Multidrug resistance	Ward	38/60	22/49	2.12 (0.98–4.58)	Trend, not significant

**Figure 2:**
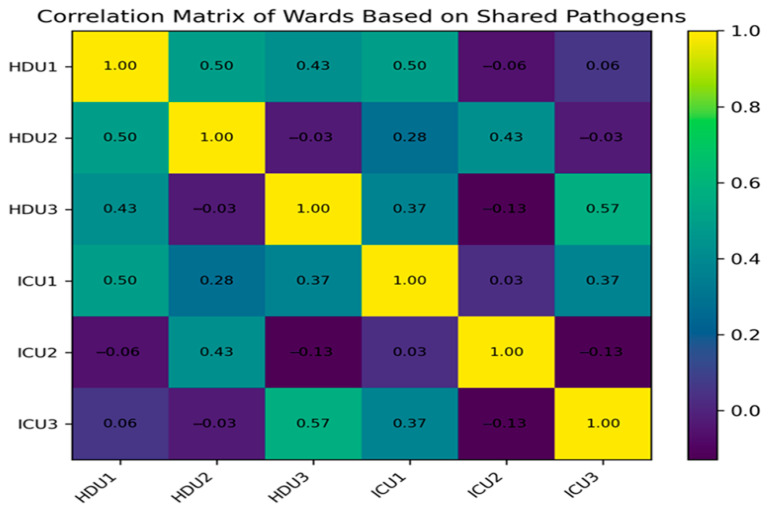
Co-occurrence matrix comparing clinical (patient) isolates with environmental isolates. Cell values indicate the number of paired isolates; diagonal cells highlight identical species recovered from both sources. The correlation matrix reflects the degree to which wards share similar species patterns. These co-occurrence relationships suggest overlapping ecological niches but do not infer directional transmission.

For each outcome (meropenem resistance, imipenem resistance, and multidrug resistance), the table shows the number of resistant isolates out of the total in each ward (ICU vs. HDU), the odds ratio with 95% confidence interval (ICU relative to HDU), and an interpretation. As shown in Table 4, meropenem resistance was significantly higher in ICU isolates compared to HDU isolates (65.0% vs. 42.9%, χ^2^ = 4.49, *p* = 0.021). In contrast, imipenem resistance was nearly identical between ICU and HDU isolates (55.0% vs. 55.1%, *p* = 0.993). Multidrug resistance (MDR, ≥3 antibiotic classes) was more frequent in the ICU (63.3% vs. 44.9% in HDUs), although this difference did not reach statistical significance (χ^2^ = 3.70, *p* = 0.054). To further examine similarities in microbial profiles between wards, we generated a correlation matrix based on shared pathogens (Figure 2). For example, HDU1 and HDU2 showed a moderately positive correlation (r = 0.50), indicating that they more frequently shared similar pathogen distributions, whereas ICU1 and HDU3 had a weaker correlation (r = 0.37), indicating less similarity. ICU2 exhibited negative correlations with several other wards, suggesting a distinct microbial community in ICU2. These correlations describe patterns of co-occurrence and similarity in species composition between units, but they do not demonstrate direction or specific routes of transmission. The patterns observed indicate the co-presence of species between units, but do not allow direct routes of transmission to be inferred.

### 3.4. Network Analysis and Co-Occurrence Patterns

**Figure 3:**
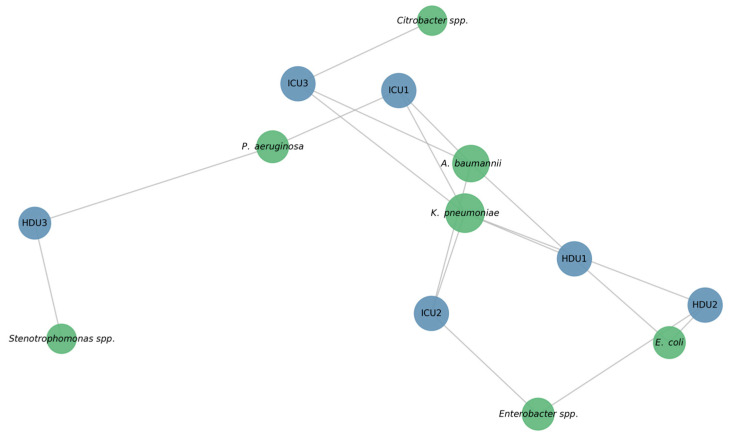
Ward–species network.

**Figure 4:**
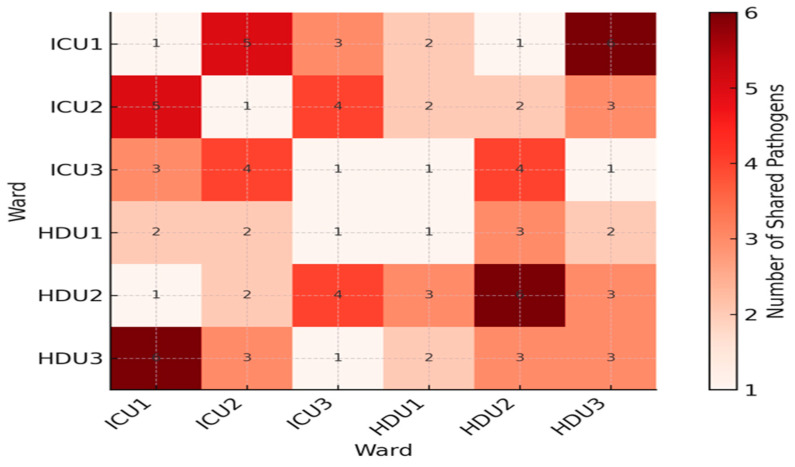
Heat map illustrating the number of distinct pathogens shared between each pair of wards. Wards are labeled on both axes. Color intensity reflects the count of shared pathogens, with red indicating higher overlap (more shared pathogens) and cooler colors indicating fewer. The highest co-occurrence (red) marks the ward pair with the most extensive pathogen sharing.

**Figure 5:**
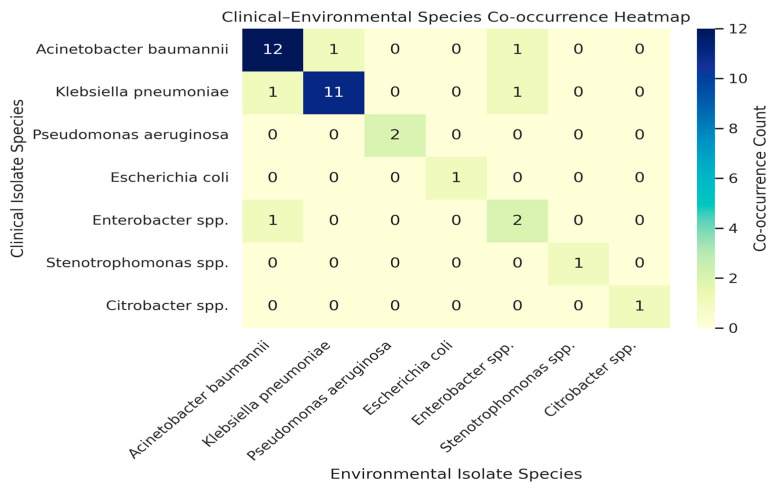
Clinical–environmental co-occurrence heatmap of Gram-negative species isolated from critical-care wards. Each cell represents the number of wards (ICUs or HDUs) in which both a clinical isolate (row species) and an environmental isolate (column species) were detected. Diagonal cells reflect species found in both patient and environmental samples within the same ward, indicating co-presence in clinical and environmental niches. Off-diagonal cells indicate co-occurrence of different species in the same ward environment. The color intensity corresponds to the frequency of co-occurrence, with darker shades indicating higher overlap. These ecological patterns indicate co-presence of species but do not, on their own, establish direct transmission pathways.

**Figure 6:**
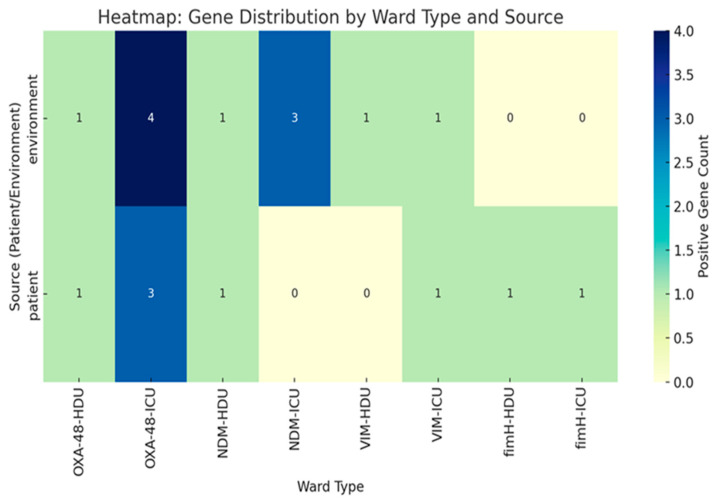
Distribution of Carbapenemase and virulence genes by ward type (HDU vs. ICU) and source (patient vs. environment). Numeric values indicate the number of gene-positive isolates.

**Figure 7:**
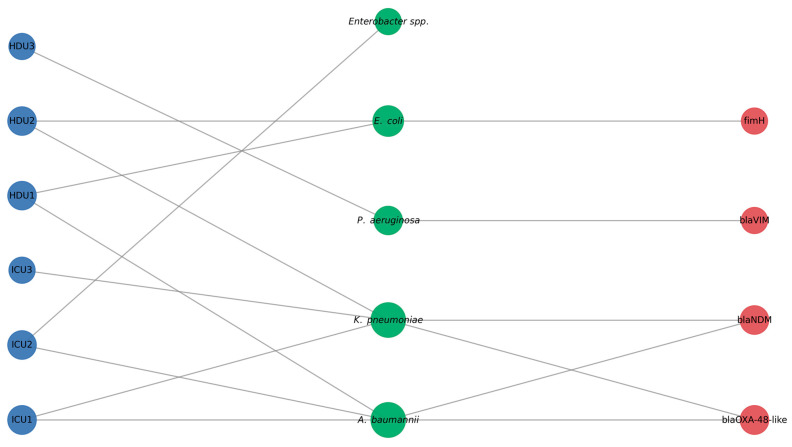
Ward–species–gene network.

To illustrate ward–pathogen relationships, we built a bipartite network linking wards and species (Figure 3). ICU and HDU acted as hubs connecting to many organisms, while *Citrobacter* spp. and *Stenotrophomonas* spp., were less connected. A heat map of distinct pathogens shared between ward pairs (Figure 4) showed ICU2 and HDU2 shared the most (six), while HDU3 and ICU3 shared the fewest A clinical–environmental co-occurrence matrix (Figure 5) showed that the same species were often found in both patient and environmental samples, especially *Acinetobacter* and *Klebsiella*, off-diagonal counts were low, indicating little cross-species overlap. A gene-distribution heat map (Figure 6) showed *bla*OXA-48 like was mainly in ICU sources and *bla*NDM in both ward types, while *bla*VIM and fimH were rare. Finally, a pathogen–gene–ward network (Figure 7) showed how resistance genes link species to ward types.

This bipartite network shows the distribution of major Gram-negative species in ICU and HDU wards. Blue nodes are hospital wards (ICU1–ICU3, HDU1–HDU3). Green nodes represent bacterial species isolated during surveillance. Node size indicates degree centrality, reflecting how frequently each ward or species is connected in the network. Edges show the presence of a species in a ward and use uniform gray lines to align with a minimalist style. The layout highlights *A. baumannii* and *K. pneumoniae* as the most widely distributed organisms. These species appear in multiple wards, underlining their dominant role among multidrug-resistant Gram-negative pathogens in critical-care settings.

This tripartite network shows connections between hospital wards, bacterial species, and carbapenemase/virulence genes detected during surveillance. Blue nodes represent wards. Green nodes represent bacterial species. Red nodes mark resistance or virulence genes (*bl*aOXA-48-like, *bla*NDM, *bla*VIM, fimH). Node size reflects degree centrality and indicates each element’s relative connectivity. Gray edges show associations between wards and species, or between species and genes. The layout highlights *A. baumannii* and *K. pneumoniae* as central species. These are linked to multiple wards and major carbapenemase genes, illustrating their critical role in the antimicrobial resistance burden of the ICUs and HDUs.

**Figure 8:**
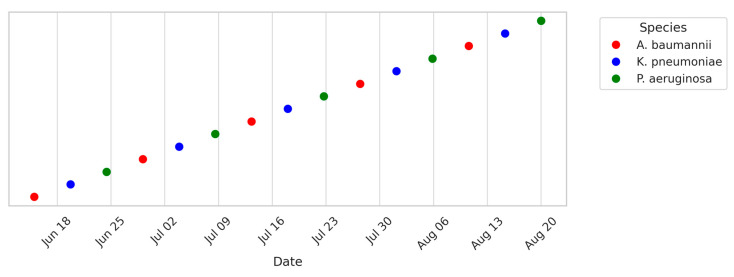
Timeline of ICU patient isolates collected between June and August 2024. Each point represents a bacterial isolate recovered from ICU patients. Symbols are color-coded by species, as shown in the legend. Clustering of *A. baumannii* (red) and *K. pneumoniae* (blue) highlights their dominance during the early surveillance phase.

**Figure 9:**
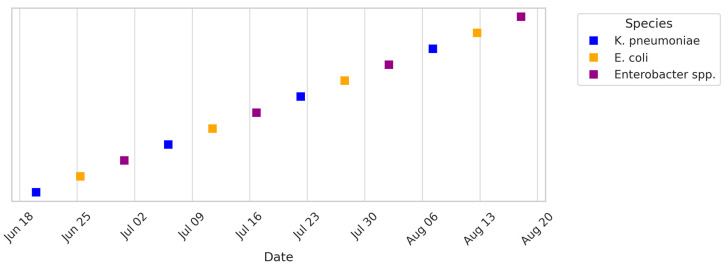
Timeline of HDU patient isolates collected between June and August 2024. Each square denotes a clinical isolate from HDU patients. Distinct colors represent different Gram-negative species, with *K. pneumoniae* and *E. coli* observed consistently across the period.

**Figure 10:**
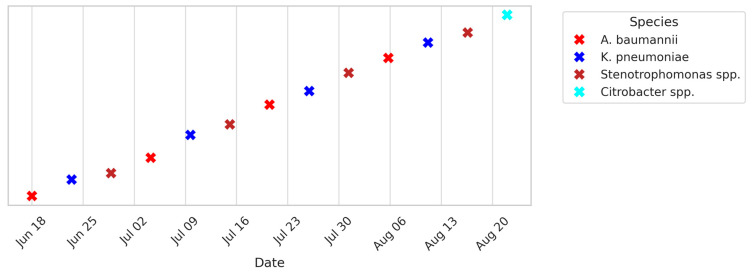
Timeline of environmental isolates collected from critical-care wards between June and August 2024. Each “X” marker represents a Gram-negative bacterial species isolated from high-touch surfaces (e.g., beds, trolleys, monitors). Colors correspond to species as shown in the legend. The timeline highlights the recurrent detection of *A. baumannii* (red) and *K. pneumoniae* (blue), mirroring trends observed in clinical isolates and underscoring environmental reservoirs as potential transmission sources. Note: ‘X’ markers and slight vertical spread were used to distinguish overlapping environmental data points for visual clarity.

To capture temporal patterns, timelines of ICU and HDU patient isolates and environmental isolates were generated (Figure 8, Figure 9 and Figure 10). These plots span June–August 2024 and show a clustering of isolates around mid-July, coinciding with a period of high patient admissions. Environmental isolates mirrored patient isolates, underscoring the dynamic interchange between patients and surroundings. Gel electrophoresis images document PCR products for *bla*NDM (621 bp), *bla*OXA-48 like (~400 bp), fimH (600 bp), and *bla*VIM (~390 bp) in (Supplementary Figures S1–S4).

## 4. Discussion

### 4.1. Key Findings

This study provides an integrated assessment of multidrug-resistant Gram-negative organisms within critical-care wards. It combines clinical isolates, environmental sampling, antimicrobial susceptibility data, and targeted gene detection. Across both ICUs and HDUs, *A. baumannii* and *K. pneumoniae* dominated the microbial landscape. This finding is consistent with global and regional reports identifying these species as major contributors to hospital-associated infections in high-dependency settings [31,32]. Their frequent recovery from surfaces underscores the well-recognized ability of MDR Gram-negative organisms—particularly *A. baumannii*—to persist in dry environments and establish long-term contamination reservoirs [33,34].

### 4.2. Antimicrobial Resistance and Carbapenemase Genes

Antimicrobial resistance patterns were severe. More than half of all isolates exhibited non-susceptibility to carbapenems and other broad-spectrum agents. These findings align with recent Pakistani and South Asian surveillance showing high carbapenem resistance among Enterobacterales and non-fermenters [35,36]. CLSI clinical breakpoints detect clinically resistant organisms but can miss early or low-level carbapenemase producers, especially OXA-48-like–producing Enterobacterales, which may remain susceptible to meropenem. ECOFFs, by contrast, separate wild-type from non–wild-type populations regardless of clinical resistance. Omitting ECOFF criteria may lead to some “stealth” carbapenemase producers being classified as susceptible, underestimating the true burden of carbapenemase-producing Enterobacterales in this cohort. This is a key limitation in regions where OXA-48-like enzymes are prevalent. The notably high colistin non-susceptibility (68.6%) should be interpreted in light of the smaller denominator (n = 51), yet still reflects an emerging regional concern reported in several ICU-based studies. Such resistance rates severely limit therapeutic options. They reinforce the need for updated local antibiograms and strengthened stewardship programs.

Resistance levels differed by ward type. ICU isolates showed higher meropenem non-susceptibility than HDU isolates. This gradient likely reflects differences in patient acuity, invasive device use, and antibiotic exposure—factors repeatedly identified as drivers of MDR proliferation in critical-care settings [37,38]. Observing this pattern in our setting supports the argument that ICU-specific antimicrobial policies and targeted infection-control measures may be particularly impactful.

Gene detection among meropenem-resistant isolates revealed a predominance of *bla*OXA-48-like, followed by *bla*NDM. This is consistent with established resistance mechanisms in Pakistan and neighboring regions [39,40]. The detection of *bla*NDM in a substantial subset also mirrors recent global trends, where *bla*NDM-producing organisms have expanded rapidly across healthcare systems [41]. Occasional detection of *bla*VIM and fimH suggests additional layers of resistance and virulence. However, interpretation of gene distribution must be limited to the meropenem-resistant subset, as PCR was not performed on susceptible isolates.

### 4.3. Clinical–Environmental Co-Occurrence and Network Insights

Network analyses and clinical–environmental co-occurrence matrices identified ICU1 and HDU2 as highly connected units. This reflects broader species diversity in these wards. These connections do not represent confirmed transmission pathways. Because whole-genome sequencing was not undertaken, our findings indicate co-presence and shared ecological patterns, rather than direct patient-to-patient or surface-to-patient transmission. Previous studies that combined patient and surface surveillance have shown that co-occurrence often precedes documented transmission events. This supports the value of maintaining this integrated approach [42,43].

The environmental contribution to the overall burden was notable. More than half of the isolates were recovered from high-touch surfaces. These findings reinforce the importance of environmental reservoirs in sustaining MDR circulation within critical-care units. The ICU and HDU follow standard protocols that include the WHO ‘Five Moments for Hand Hygiene’, twice-daily cleaning of high-touch surfaces with quaternary ammonium disinfectants, and terminal decontamination after patient discharge. However, variations in adherence, workflow pressures, and the persistence of MDR Gram-negative organisms on surfaces may reduce the effectiveness of these measures. These findings highlight the importance of strengthening compliance monitoring, optimizing environmental cleaning practices, and prioritizing high-risk surfaces in critical-care settings. Prior work has shown that persistent contamination of equipment, bed rails, trolleys, and ventilator interfaces can facilitate the maintenance of difficult-to-eradicate MDR populations [26,44]. In our study, repeated detection of *A. baumannii* and *K. pneumoniae* in both clinical and environmental samples suggests that patient care areas operate as closely connected microbial ecosystems. While genomic analyses would be required to confirm directionality, the observed ecological overlap highlights the need for systematic cleaning protocols, equipment cohorting, and focused environmental audits.

The molecular findings, combined with high phenotypic resistance, also underscore the urgency of expanding access to newer therapeutics. Agents such as sulbactam–durlobactam and cefiderocol have shown activity against carbapenem-resistant *A. baumannii* and *K. pneumoniae* in international evaluations [45,46]. In settings where colistin resistance is rising, such options may provide essential alternatives. Their adoption must be guided by robust stewardship frameworks.

### 4.4. Strengths and Limitations

This study’s strengths include the combined use of clinical and environmental surveillance, the incorporation of molecular assays, and the application of network modeling to identify potential contamination hubs. Limitations include its cross-sectional design, single-center setting, and molecular testing restricted to meropenem-resistant isolates. Despite these limitations, the findings provide a blueprint for facility managers and policy makers to identify high-risk reservoirs and implement surveillance-driven infection prevention measures, even where resources are constrained.

## 5. Conclusions

This study demonstrates a substantial burden of multidrug-resistant *A. baumannii* and *K. pneumoniae* across critical-care wards. Environmental surfaces are major reservoirs, in addition to patient isolates. Carbapenem and colistin non-susceptibility were frequent. This underscores the increasingly constrained treatment options for critically ill patients. Resistance was more pronounced in ICU settings, reflecting higher antibiotic pressure and device use. PCR clarified the distribution of key carbapenemase genes. However, the absence of genomic sequencing limits interpretation to ecological overlap rather than confirmed transmission. These findings highlight the need for strengthened clinical–environmental surveillance, targeted decontamination of high-touch surfaces, and reinforced antimicrobial stewardship. Prioritizing contamination hotspots and monitoring evolving carbapenemase patterns will be essential for reducing the spread of resistant organisms in high-risk hospital settings.

## Figures and Tables

**Table 1:** Antimicrobial susceptibility profile of 109 Gram-negative ICU/HDU isolates. Breakpoints were interpreted according to CLSI M100 (2024). For analysis, non-susceptible was defined as intermediate or resistant versus susceptible. Colistin was tested only in a reduced subset of isolates (*n* = 51) because of plate availability and isolate viability.

Antibiotic	Tested (*n*)	Resistant *n* (%) [95% CI]	Sensitive *n* (%)	Intermediate *n* (%)
Imipenem	109	60 (55%) [45.4–64.2]	48 (44%)	1 (0.9%)
Meropenem	109	60 (55%) [45.4–64.2]	49 (45%)	0 (0%)
Piperacillin–tazobactam	109	62 (56.9%) [47.3–66.0]	45 (41.3%)	2 (1.8%)
Ceftazidime	107	62 (57.9%) [48.2–67.1]	45 (42.1%)	0 (0%)
Ciprofloxacin	108	62 (57.4%) [47.8–66.6]	46 (42.6%)	0 (0%)
Amikacin	106	52 (49.1%) [39.4–58.7]	54 (50.9%)	0 (0%)
Colistin *	51	35 (68.6%) [54.1–80.9]	8 (15.7%)	8 (15.7%)

* Colistin susceptibility testing was performed only on a targeted subset of Gram-negative isolates using broth microdilution MIC; therefore, the denominator for colistin (*n* = 51) differs from other antibiotics.

**Table 2:** Distribution of Gram-negative organisms by ward among 109 isolates recovered from ICU and HDU patients.

Bacterial Species	HDU (*n* = 49)	ICU (*n* = 60)	Total (*n* = 109)
*Acinetobacter baumannii*	15 (30.6%)	25 (41.7%)	40 (36.7%)
*Klebsiella pneumoniae*	12 (24.5%)	25 (41.7%)	37 (33.9%)
*Pseudomonas aeruginosa*	9 (18.4%)	3 (5%)	12 (11%)
*Escherichia coli*	7 (14.3%)	2 (3.3%)	9 (8.3%)
*Enterobacter* spp.	5 (10.2%)	3 (5%)	8 (7.3%)
*Stenotrophomonas* spp.	1 (2%)	1 (1.7%)	2 (1.8%)
*Citrobacter* spp.	0 (0.0%)	1 (1.7%)	1 (0.9%)
Total	49 (100%)	60 (100%)	109 (100%)

**Table 4:** Comparison of meropenem resistance, imipenem resistance and multidrug resistance (MDR) between HDU and ICU isolates (*n* = 109). Breakpoints were interpreted according to CLSI M100 (2024), and non-susceptible was defined as intermediate or resistant versus susceptible.

Parameter	HDU (*n* = 49)	ICU (*n* = 60)	Test Used	*p*-Value
Meropenem resistant	21 (42.9%)	39 (65%)	χ^2^ test	0.021
Imipenem resistant	27 (55.1%)	33 (55%)	χ^2^ test	0.993
MDR (≥3 antibiotic classes)	22 (44.9%)	38 (63.3%)	χ^2^ test	0.054

MDR definition: Multidrug resistance was defined as resistance to three or more antibiotic classes [30]. Note: Fisher’s exact test was not required for any of the above comparisons because all expected cell counts were ≥5; therefore, the chi-square test was appropriate.

## Data Availability

The original contributions presented in this study are included in the article/Supplementary Material. Further inquiries can be directed to the corresponding author.

## Supplementary Materials

The following supporting information can be downloaded at: https://www.mdpi.com/article/10.3390/microorganisms13122762/s1, Figure S1: Agarose gel showing PCR products of *bla*NDM (621 bp). L = ladder; numbers correspond to isolate IDs; NC = negative control; Figure S2: Agarose gel showing PCR products of *bla*OXA-48 (~400 bp). L = 100 bp ladder; NC = negative control; Figure S3: Agarose gel illustrating PCR products of fimH (600 bp) and blaOXA-48 (400 bp). Dual bands indicate co-detection of the virulence gene fimH and the carbapenemase gene; Figure S4: Agarose gel showing PCR products of *bla*VIM (~390 bp). L = 100 bp ladder; NC = negative control; Table S1: Full multivariable logistic regression model for predictors of meropenem non-susceptibility (*n* = 109).

## Abbreviations

AST	Antimicrobial susceptibility testing
ATCC	American Type Culture Collection
blaNDM	New Delhi metallo-β-lactamase gene
blaOXA-48	Oxacillinase-48-like carbapenemase gene
blaVIM	Verona integron-encoded metallo-β-lactamase gene
CLSI	Clinical and Laboratory Standards Institute
FDA	U.S. Food and Drug Administration
HDU	High-dependency unit
ICU	Intensive care unit
MDR	Multidrug resistant
MIC	Minimum inhibitory concentration
NDM	New Delhi metallo-β-lactamase
OR	Odds ratio
OXA-48	Oxacillinase-48-like carbapenemase
VIM	Verona integron-encoded metallo-β-lactamase
