# Supplementary material for: Patients and Surfaces: Integrated Clinical–Environmental Surveillance of MDR Gram-Negative Bacteria in Critical-Care Units (Karachi, 2024–2025)
Source: Microorganisms. 2025 Dec 4;13(12):2762. doi: 10.3390/microorganisms13122762 (PMC12735442; doi:10.3390/microorganisms13122762)
Supplement: Supplementary file 1 [file microorganisms-13-02762-s001.zip › microorganisms-4007749-supplementary.pdf]

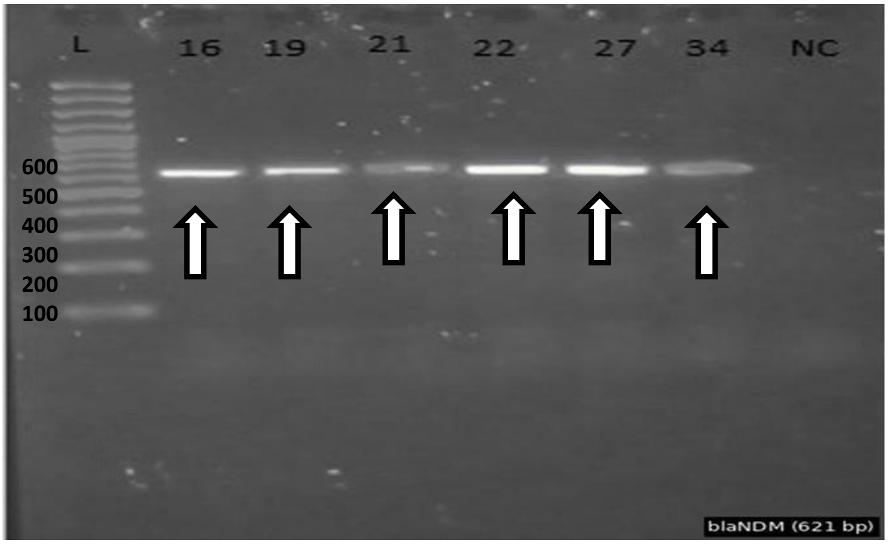

Figure S1 – Agarose gel showing PCR products of blaNDM (621 bp). L = ladder; numbers correspond to isolate IDs; NC = negative control.

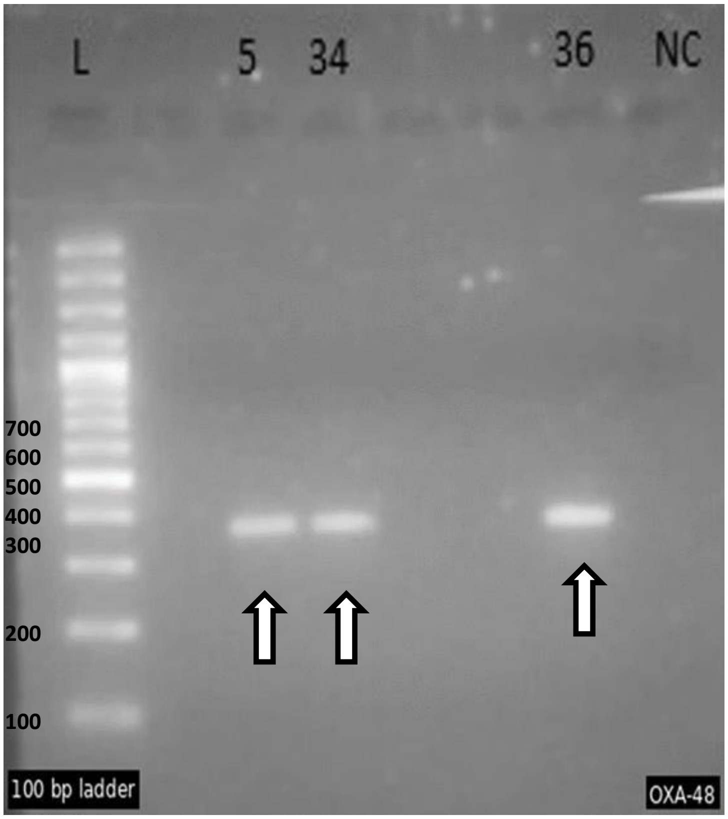

Figure S2 – Agarose gel showing PCR products of blaOXA-48 (~400 bp). L = 100 bp ladder; NC = negative control.

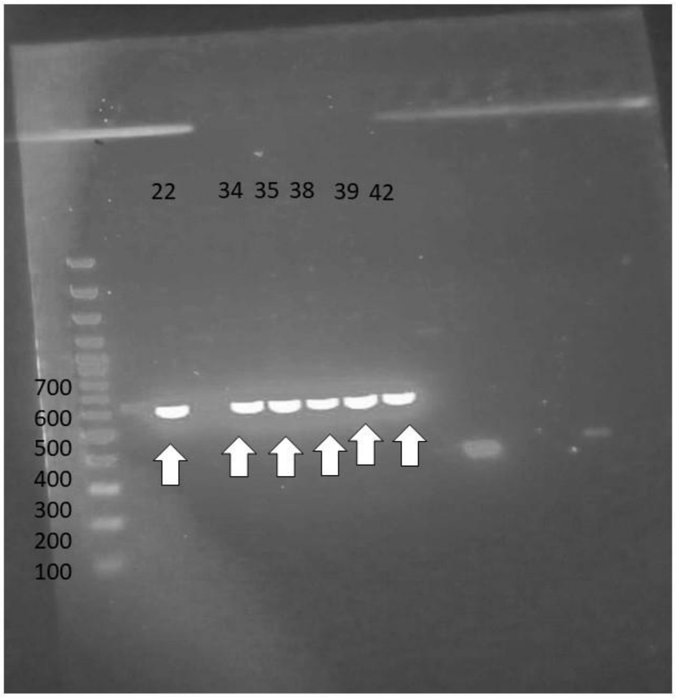

Figure S3 – Agarose gel illustrating PCR products of fimH (600 bp) and blaOXA-48 (400 bp). Dual bands indicate co-detection of the virulence gene fimH and the carbapenemase gene.

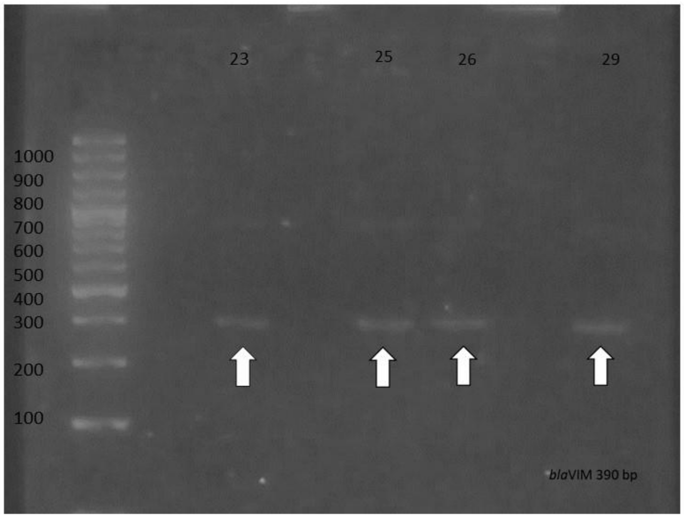

Figure S4 – Agarose gel showing PCR products of blaVIM (~390 bp). L = 100 bp ladder; NC = negative control.

Supplementary Table S1. Full multivariable logistic regression model for predictors of meropenem non-susceptibility (n = 109).

Variable	β Coefficient	Standard Error	Odds Ratio (OR)	95% CI (OR)	p-value
**Intercept**	-0.842	0.621	–	–	0.172
**Ward Type (ICU vs HDU)**	0.928	0.402	**2.53**	1.15 – 5.58	**0.021**
**Source (Environmental vs Clinical)**	0.311	0.378	1.36	0.65 – 2.82	0.410
**Species (ref: *K. pneumoniae*)**	–	–	–	–	–
• A. baumannii	0.741	0.462	2.10	0.87 – 5.08	0.095
• P. aeruginosa	0.532	0.687	1.70	0.44 – 6.48	0.445
• E. coli	-0.214	0.762	0.81	0.18 – 3.57	0.780
• Enterobacter spp.	-0.132	0.813	0.87	0.18 – 4.17	0.867
**Month (ref: June)**	–	–	–	–	–
• July	0.412	0.487	1.51	0.58 – 3.89	0.393
• August	0.692	0.521	1.99	0.71 – 5.60	0.190

Model Diagnostics:

- **Number of observations:** 109
- **Pseudo-R^2 (McFadden):** 0.18
- **χ^2 (model):** 14.87, df = 8, p = 0.060
- **Clustering:** Robust SEs clustered by ward

Interpretation:
ICU location remained the only statistically significant predictor of meropenem non-susceptibility (OR = 2.53, p = 0.021), after adjusting for species, source, and month of isolation.
